# Colorimetric Paper Sensor for Food Spoilage Based on Biogenic Amine Monitoring

**DOI:** 10.3390/bios13010126

**Published:** 2023-01-11

**Authors:** Maria Maddalena Calabretta, Denise Gregucci, Riccardo Desiderio, Elisa Michelini

**Affiliations:** 1 Department of Chemistry “Giacomo Ciamician”, Alma Mater Studiorum-University of Bologna, Via Selmi 2, 40126 Bologna, Italy; 2 Center for Applied Biomedical Research (CRBA), IRCCS St. Orsola Hospital, 40138 Bologna, Italy; 3 Health Sciences and Technologies Interdepartmental Center for Industrial Research (HSTICIR), University of Bologna, 40126 Bologna, Italy

**Keywords:** biogenic amines, paper sensor, food spoilage, food safety, smartphone, colorimetric detection, genipin

## Abstract

Biogenic amines (BAs), nitrogenous molecules usually present in different foods, can be considered an indicator of freshness and food quality since their amount increases during food spoilage. Their detection, possibly in real time via the use of smart packaging, is therefore of crucial importance to ensure food safety and to fulfill consumers’ demand. To this end, colorimetric sensors are considered one of the most feasible solutions. Here, we report a user-friendly colorimetric sensing paper able to detect BAs via the naked eye. The sensing molecule is the aglycone genipin, a natural cross-linking agent extracted from gardenia fruit, able to bind BAs producing water-soluble blue pigments. The paper sensor was applied to chicken meat quality monitoring and a quantitative analysis was performed with image acquisition via a smartphone camera, achieving a limit of detection equivalent to 0.1 mM of putrescine. The suitability of the BA sensing paper was assessed by integrating the sensor into smart packaging and analyzing commercial chicken meat samples stored at different temperatures; the results of the sensor paralleled the “best before date” indicated on the label, confirming the potential applicability of the sensor as a smart label.

## 1. Introduction

One of the current challenges, which is recognized as a priority by the United Nations, is to ensure food safety, security and sustainability to feed the expected population of 10 billion persons in 2050.

It has been estimated that, each year, there are approximately 420,000 deaths caused by unsafe food [1]. In this scenario, the reduction of food waste and monitoring of food quality and safety are of the utmost importance. The food industry is always looking for new methodologies to monitor the quality and freshness of food [2], the authenticity and potential adulterations of food [3,4] and toxins and other contaminants [5,6]. With the increase in consumers’ desire to actively monitor food quality, the food packaging industry is facing new challenges in monitoring the entire food distribution chain and preventing food spoilage and counterfeited food products.

One of the main causes of food spoilage is the growth of microorganisms originally present in the food or arising from external contaminations, leading to the formation of undesirable by-products. These by-products may change the appearance, texture, taste and smell of food, making it unfit and unhealthy for human and animal consumption [7].

Biogenic amines (BAs), nitrogenous molecules usually present in different foods, can be considered an indicator of freshness and food quality since their amounts increase during food spoilage. Normal levels of BAs do not pose health risks but, during food processing and inadequate temperature storage conditions, their concentration can increase due to the bacterial decarboxylation of amino acids or transamination of aldehydes and ketones, leading to important human allergic reactions, digestive problems and food poisoning [8,9,10].

While food poisoning of microbial origin can be prevented simply by heating food, BAs are thermoresistant molecules and are thus stable even after cooking [11].

Several analytical techniques have been developed to selectively detect BAs, such as cadaverine and putrescine, even at low concentrations, most of them relying on chromatography and enzyme-based assays [12]. Nevertheless, these methods cannot be deployed in the field and still require complex instrumentation or skilled personnel. More recently, other methods based on fluorescent probes [13], modified lanthanide metal–organic frameworks [14], functionalized single-walled carbon nanotubes [15], or colorimetric sensor arrays have been proposed to detect BAs associated with food spoilage [16]; however, the possibility to integrate these probes and sensors into food packaging remains to be demonstrated [17,18]. While several biosensors for microbe detection have been developed based on microfluidic chips [19], lateral flow assays and surface-enhanced Raman scattering (SERS), fewer methods have been reported for the detection of BAs having the potential to be integrated into smart packaging [20,21].

Simple and inexpensive colorimetric sensors are an alternative to sophisticated, costly and time-consuming instrumental analytical methods that require professional personnel. Colorimetric user-friendly lab-on-paper sensors [22], arrays [23] and plastic film indicators [24] have been developed for food spoilage in perishable meat or seafood products [25].

Although colorimetric sensors based on single or multiple chromogenic reagents have been developed, a low color resolution or complicated design, as well as difficulties in handling and the incorporation of chemical dyes into the carrier system, hamper their use. In addition, since these sensors must be placed in contact with food, the use of safe or even edible molecules should be ensured for future uptake in the food packaging market. A cost-effective and environmentally friendly alternative is represented by natural pigments widely used as pH indicators that can be integrated into packaged products [26,27,28].

To provide a green and user-friendly platform, we developed a colorimetric paper sensor for the in situ detection of BAs and evaluation of food freshness and spoilage. The proposed sensor exploits the reactivity of the iridoid genipin, able to bind, in the presence of molecular oxygen, primary amines present in amino acids and proteins to produce water-soluble blue pigments [29,30]. Genipin was entrapped into a paper sensor to provide a disposable device for BAs, suitable for integration into smart packaging. The paper sensor was applied to the analysis of chicken meat quality monitoring, allowing the detection of BAs with adequate reproducibility and accuracy.

## 2. Materials and Methods

### 2.1. Chemicals, Reagents and Instrumentation

Genipin (methyl (1S,2R,6S)-2-hydroxy-9-(hydroxymethyl)-3-oxabicyclo [4.3.0]nona-4,8-diene-5-carboxylate), putrescine (1,4-Diaminobutane), agarose for molecular biology and all other chemical reagents were from Sigma-Aldrich (St. Louis, MO). Whatman 1 CHR cellulose chromatography paper was from GE Healthcare (Chicago, IL, USA) and was used as support for the design of the colorimetric sensing paper. OnePlus 6T (OnePlus, Shenzhen, China) was used for colorimetric signal acquisitions. A Phaser 8400 office wax printer (Xerox, Norwalk, CT, USA) was used for wax printing.

### 2.2. Experimental Design and Method Optimization in Liquid Format

The first optimization was aimed at defining the optimized genipin concentration able to react with BAs. To this end, two different concentrations of genipin (5.0 mg/mL and 2.5 mg/mL) were tested. A calibration curve was obtained in a clear microtiter 96-well plate, adding 40 µL of 5.0 mg/mL genipin solution and a 40 µL volume of putrescine solution (concentration range from 0 to 5 mM) and incubating it at room temperature (+25 °C) for 0.5, 1, 2, 4, 5 and 18 h. A OnePlus 6T smartphone was used to acquire colorimetric signals at the different incubation times, and ImageJ v. 1.53t software was used to analyze the image. All the measurements were performed in triplicate and repeated at least three times.

### 2.3. Method Optimization on Paper

Genipin stock solution (22 mM) was prepared by dissolving 5.0 mg of genipin in 1.0 mL of deionized water (ddH_2_O) in an ultrasonic bath [31]. To create a paper sensor for BAs, different immobilization strategies were explored, i.e., adsorption and entrapment, to integrate genipin in a paper format.

**Figure 1:**
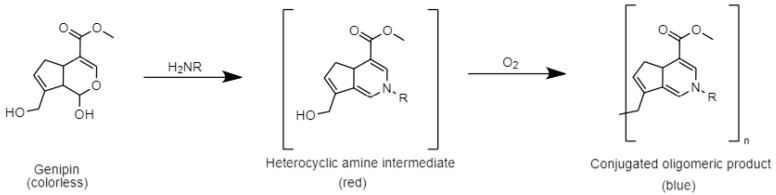
Schematic mechanism of genipin reaction in presence of BAs.

A configuration composed of 24 circular areas (an 8 × 3 array of 5-mm-diameter wells) for a putrescine calibration curve, each one representing a microwell in which the colorimetric reaction occurs (Figure 1), was first designed with PowerPoint (Microsoft, Redmond, WA, USA) and printed onto Whatman 1 CHR chromatography paper using an office wax printer. Hydrophobic areas were created by heating the waxed pattern for 2 min at 80 °C.

For the adsorption procedure, a 20 µL volume of the 22 mM genipin stock solution was added to a 5-mm-diameter paper well and left to dry for 30 min at room temperature [32]. This volume was selected because it provided homogeneous absorption within the hydrophilic area of the paper well. For the entrapment, an optimized protocol previously reported by Lopreside et al. [6] was selected to integrate the genipin on the paper. Briefly, a solution of 1.5% *w/v* agarose was prepared by heating in a water bath at 100 °C for 2 h, and then the solution was allowed to cool down to a temperature of around 40–50 °C before adding a 5 mg/mL genipin solution (1:1 ratio). Then, a 20 µL volume was dispensed on the previously printed circular wells. Putrescine calibration curves were obtained by the addition of a 20 μL volume of different solutions (concentration range from 0.06 to 5.0 mM) and left to incubate for 0.5, 1.0, 2 and 3 h at room temperature (25 °C). All measurements were performed in triplicate and repeated at least three times.

### 2.4. Reflectance Signal Acquisition and Data Analysis

Reflectance measurements were acquired with a OnePlus 6T smartphone (OnePlus, Shenzhen, China), equipped with a dual integrated camera, composed of a primary sensor (16 MP Sony Exmor RS IMX 519, BSI CMOS ½.600 color sensor with 1.22 μm pixels, ƒ/1.7 aperture) and a secondary sensor (20 MP Sony Exmor RS IMX 376 K, BSI CMOS ½.800 color sensor with 1.0 μm pixels, ƒ/1.7 aperture). Colorimetric signals were analyzed with ImageJ software (v. 1.53t), National Institutes of Health, Bethesda, MD, USA). Colorimetric signals were evaluated by brightness analysis over the circular region of interest (ROI) area defined in correspondence to the sensor wells, as previously described [33]. GraphPad Prism v.8 software (GraphPad Software, LaJolla, CA, USA) was used to fit the brightness data for the creation of the BA calibration curve using second-order polynomial (quadratic) non-linear regression. The limit of detection (LOD) was calculated as the mean value of the blank (ddH_2_O) minus three times the standard deviation of the control sample (ddH_2_O). The limit of quantification (LOQ) was calculated as the mean value of the blank (ddH_2_O) minus ten times the standard deviation of the control sample (ddH_2_O). All measurements were performed in triplicate and repeated at least three times. For BA monitoring in chicken meat samples, the colorimetric signals at +4 °C and room temperature (+25 °C) obtained at day 0 were normalized as 100% of the signal.

### 2.5. Design of Colorimetric BA Sensing Paper

The suitability of the developed paper sensor for the analysis of real samples was evaluated by detecting BAs in chicken meat samples. To this end, a sensor pattern was designed with PowerPoint (Microsoft, Redmond, WA, USA) and printed onto Whatman 1 CHR chromatography paper using a wax printer. To create hydrophobic areas, the waxed pattern was heated for 2 min at 80 °C, allowing it to diffuse throughout the paper. The final configuration of the colorimetric BA sensing paper consisted of a T-shaped sensor with three circular wells (diameter of 5 mm), two of which were used as chromatic indicators of the presence (HARMFUL well) or absence (SAFE well) of BAs and were blue (R = 68, G = 114, B = 195) and white (R = 255, G = 255, B = 255), respectively. The third well (TEST well) was the sample well for detecting BAs in real samples.

### 2.6. Real Sample Analysis

Fresh boneless chicken fillet samples were obtained from a local market (Coop, Bologna, Italy), cut into twenty pieces of approximately 50 g, and each piece placed into a petri dish. Sensor responsiveness was assessed by integration of the BA sensing paper in each chicken sample, sealed with cling film. To mimic real-life conditions in which chicken meat can be consumed even a few days after purchase, the monitoring of food spoilage was carried out at room temperature (+25 °C) and at +4 °C for a 3-day period. All experiments were performed in triplicate and repeated at least three times. The image was acquired with the OnePlus6T smartphone camera and analyzed with ImageJ software and compared to those obtained at day 0.

### 2.7. Stability Studies

Sensor stability was investigated for two weeks by measuring the colorimetric signals of the BA sensing paper stored at +4 °C. To this end, a series of BA sensing papers were obtained following the procedure reported in Section 2.5, followed by storage in a petri dish and sealing with cling film, used to preserve the food; samples were stored for up to two weeks at +4 °C. The sensor responsiveness was assessed by the addition of 20 μL of putrescine (5.0 mM) with an incubation period of 3 h at room temperature (25 °C). Colorimetric signals were acquired with a smartphone camera (OnePlus 6T), analyzed with ImageJ software, and compared to those obtained at day 0, i.e., with newly produced sensors.

## 3. Results and Discussion

In this work, we developed a low-cost disposable paper sensor for BA detection, with an estimated cost per sample of approximately 0.3 eurocents, exploiting the capability of genipin, a natural colorless iridoid from gardenia fruit, to react with BAs, considered as markers of food spoilage.

Exploiting a double condensation reaction, genipin reacts with BAs, producing an oxidatively unstable heterocyclic amine intermediate that further undergoes oxidative oligomerization/polymerization in the presence of molecular oxygen, resulting in a brilliant blue conjugated oligomeric product (Figure 1), visible to the naked eye [29].

### 3.1. Method Optimization in Liquid and Paper Format

**Figure 2:**
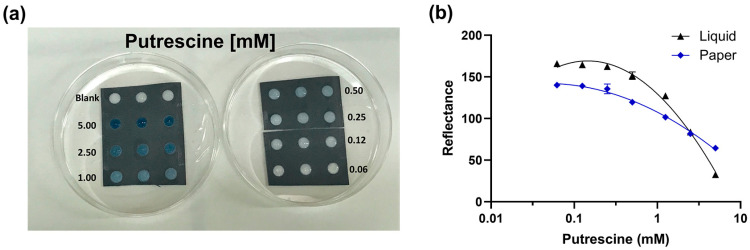
(**a**) Picture of the putrescine calibration curve (concentration range from 0.06 to 5.0 mM) obtained after 3 h of incubation at room temperature in paper format; (**b**) comparison of putrescine calibration curves (concentration range from 0.06 to 5.0 mM) in liquid (black line) and paper (blue line) format after 3 h of incubation at 25 °C. Error bars are present but not visible due to the minimum variation in reflectance signals obtained between replicates.

Preliminary experiments involving different concentrations of genipin (2.5 and 5.0 mg/mL) and incubation times (from 0.5 to 18 h) were assessed to identify the experimental conditions providing the highest sensitivity in the detection of BAs. Putrescine, which is present at concentrations from 0.5 to 383 mg/kg in meat [10], was used as a model analyte of BA; putrescine solutions (from 0.05 to 10 mM) were incubated from 0.5 to 18 h with genipin at 5.0 mg/mL (Series A) and 2.5 mg/mL (Series B). After overnight incubation at room temperature, a proportional correlation between genipin and putrescine was obtained only in Series A (Figure S1) in the concentration range from 0.05 to 5.0 mM. As shown in Figure 2a and Figure S2, the presence of BAs led to a change in color, visible to the naked eye, from white to blue upon 3 h of incubation at 25 °C. By analyzing the images obtained with the OnePlus6T smartphone using the ImageJ software, a calibration curve in liquid for putrescine was obtained with a limit of detection (LOD) of 0.09 mM (Figure 2b, black line).

After assessing the suitability of the liquid assay format to detect putrescine, we moved on to optimize the reaction conditions in paper format. To this end, a ready-to-use solid support sensitive to BAs was developed by immobilizing genipin on paper. Wells with a diameter of 5 mm were selected to provide a uniform reflectance signal [32]. Adsorption and entrapment methods were investigated to implement the assay in a portable paper format. In the biosensing field, physical adsorption is generally considered the most convenient strategy to achieve non-covalent biomolecule immobilization, especially for enzymes. In the entrapment strategy, a polymeric network is created to capture and retain biomolecules, allowing samples, substrates or products to easily diffuse. Both immobilization procedures provide non-covalent attachment, thus retaining the activity of the biomolecules. As reported in Figure S3, we compared the putrescine calibration curves obtained with genipin adsorbed and entrapped on paper, and the adsorption procedure did not allow uniform genipin distribution, as confirmed by the colorimetric signals only in the peripheral region of the microwells (data not shown). This caused a loss in sensitivity, with an LOD approximately one order of magnitude higher (1.0 mM) than that obtained with the entrapment method (LOD: 0.1 mM). As reported previously by Gu Z. et al., agarose is able to bind to filter paper fibers with the creation of a hydrogel-coated region characterized by relatively slow adsorption of liquid [34]. Therefore, the use of agarose could facilitate the interaction between genipin and the sample.

As shown in Figure 2a, different concentrations of putrescine (concentration range from 0.06 to 5.0 mM) were incubated in the paper format and, after 3 h of incubation (at 25 °C), a color change from white to blue was observed due to the presence of BAs (Figure 2a). In colorimetric paper-based analytical devices, color spots are observed by reflection. The image obtained with the OnePlus6 smartphone camera was analyzed with ImageJ software, and a calibration curve in paper format was obtained and compared with that obtained in liquid format (Figure 2b), showing similar performance (LODs for putrescine of 0.09 and 0.1 mM in liquid and paper format, respectively). Although slightly higher sensitivity was found in liquid format, the results confirmed the suitability of integrating, with high efficiency, the proposed system into smart packaging for the quantitative detection of BAs in food samples. When compared to a recently developed transcription factor-based biosensor for putrescine [35], our sensor showed a significantly lower LOD for putrescine (0.09 mM in liquid format vs. 5.37 mM) and reduced response time (0.5 h vs. 1 h). A LOQ of 0.39 mM was also obtained in paper format.

### 3.2. Design and Fabrication of BA Paper Sensor

After the optimization of the reaction conditions in paper format, a user-friendly configuration of the sensor was fabricated with the possibility of inclusion into smart packaging. To this end, a T-shaped sensing paper was developed, providing a disposable device for detecting BAs in food samples, easily readable by consumers.

**Figure 3:**
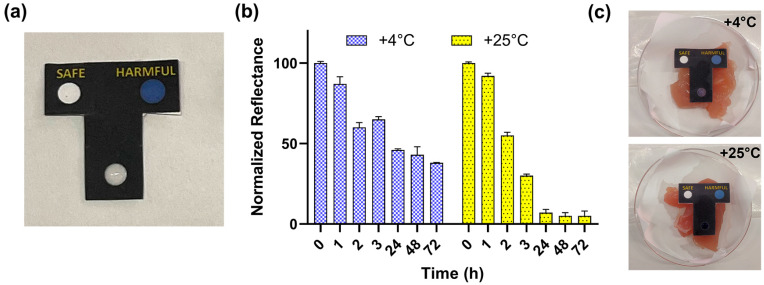
(**a**) BA sensing paper design and (**b**) BA monitoring in chicken meat samples stored at +4 °C and room temperature (+25 °C) for a 3-day period; (**c**) pictures of BA paper sensing in chicken meat samples after 24 h of storage at +4 °C and +25 °C.

The colorimetric BA sensing paper consisted of three circular wells with a diameter of 5 mm, two of which were used as chromatic indicators of the presence (HARMFUL well) or absence (SAFE well) of BAs. The third well was the sample well for detecting BAs developed in real samples (Figure 3a), where genipin was entrapped as described in the Material and Methods (Section 2.3). We decided to design the proposed system in a paper-based format since the well-known green properties of paper allow its use in close contact with food samples, enabling the passive transport of liquids; in addition, paper is compatible with many chemicals and is economical and sustainable.

Another important feature of the proposed BA sensing paper is related to the sustainable method and the ease of production in any laboratory (only an office wax printer was required as equipment). Moreover, the sensor can be discarded after use without safety concerns, as genipin is a natural product with no toxic properties, widely used, also in the United States, as a natural color additive [36].

### 3.3. Detection of BAs in Real Chicken Meat Samples

To demonstrate the suitability of the BA sensing paper for detecting food spoilage in real situations, we integrated the BA sensing paper into the packaging of chicken meat samples and evaluated its response after storage at room temperature (+25 °C) or at +4 °C for up to 3 days, corresponding to the expiration date on the label. Colorimetric images were acquired at 1, 2, 3, 24, 48 and 72 h, analyzed with ImageJ software, as described in the Materials and Methods. As shown in Figure 3b, no significant differences in BA formation at +25 °C and +4 °C were observed after 2 h, and lower brightness signals of 35% and 70% were obtained after 3 h of incubation at +4 °C and 25 °C, respectively, reaching 54% and 93% after 24 h (Figure 3c). Unlike enzymatic biosensors, in which the temperature greatly affects the catalytic activity of the enzyme and thus influences the biosensor’s performance, in the proposed sensor, relying on the natural crosslinking agent genipin, the temperature does not interfere with the sensor’s performance, while it has a well-known influence on the formation of biogenic amines during food spoilage [37,38].

Such a paper sensor integrated into the packaging provides rapid and instrument-free information about food freshness. The end-user simply compares the sensor color with the two printed controls, i.e., SAFE and HARMFUL, in the T-shaped sensor. In this configuration, the threshold corresponds to the blue well, meaning that the meat is no longer safe to eat. However, further work will be conducted to provide the consumer with a scale with different levels of warning in order to avoid unnecessary food waste. This will require the use of a smartphone as a detector and a dedicated app to guide the consumer towards the most sustainable and healthier choices. According to the European Food Safety Authority (EFSA), putrescine, together with cadaverine, is one of the most common biogenic amines found in food, with higher concentrations in fermented dairy products such as cheese (up to 1560 mg/kg) and fish (up to 337 mg/kg) [8]. Therefore, the availability of sensors targeting these targets, at present not commercially available, is highly valuable. Other reflectometric sensors have been reported, mostly based on an array of sensors, including, among others, acid–base indicators, porphyrins and nanomaterials [39]. An interesting work was reported by Bueno et al. based on a plastic-based device to discriminate three amines relying on five pH indicators; however, a chemometric analysis combined with smartphone detection was necessarily required to provide accurate results [40].

### 3.4. Stability

**Figure 4:**
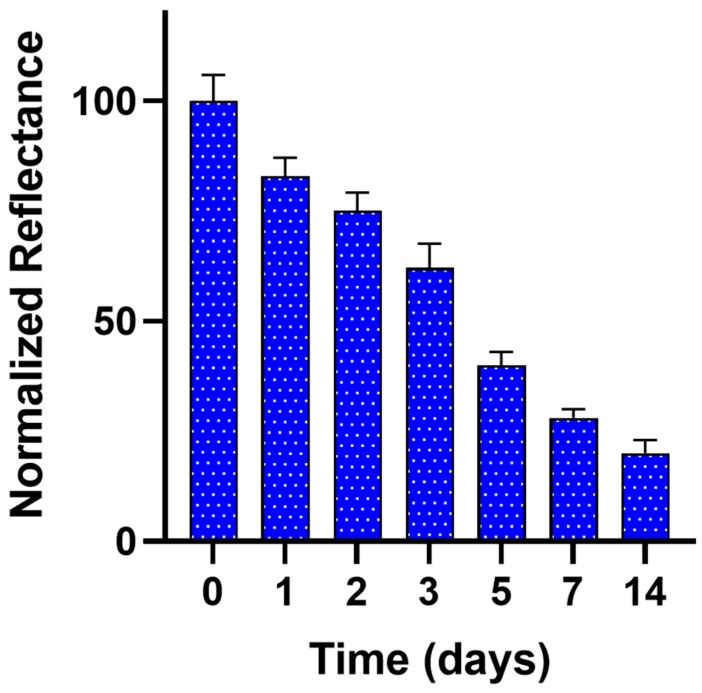
Stability of the BA sensing paper stored at +4 °C. For each measurement, three sensing papers were tested following the procedure reported in Section 2.7. Colorimetric measurements were obtained with a OnePlus 6T and analyzed with ImageJ software. Reflectance signals are normalized with respect to day 0.

As concerns the sensor’s stability, Figure 4 shows the colorimetric signals measured for BA sensing papers stored in cling film at +4 °C for two weeks. The colorimetric measurements were performed as reported in the optimized experimental protocol, normalizing the reflectance signals to the values obtained immediately after the production of our BA sensing paper (day 0). Storage of the sensing paper at +4 °C resulted in a decrease in responsiveness of approximately 60 ± 5% and 80 ± 3% after 5 and 14 days, respectively.

We investigated the reproducibility by analyzing the same sample on consecutive days using the paper sensor produced on the same day and maintained in use up to +4 °C as the optimal storage condition. Concerning the repeatability, we obtained a relative standard deviation (RSD = 5) of 10.5% for the BA sensing paper sensor produced on the same day and used for detecting an intermediate concentration of putrescine (1.0 mM).

## 4. Conclusions

We presented a new colorimetric paper-based sensor that provides a very rapid and straightforward analysis of the BAs produced in food, by exploiting genipin’s double condensation reaction with BAs in the presence of molecular oxygen, resulting in a brilliant blue conjugated oligomeric product visible to the naked eye.

The paper-based sensor described here allows quantitative measurements of BAs using small volumes of samples and, thanks to the high intensity of the colorimetric signal, enables quantitative smartphone-based detection. The BA sensing paper’s suitability was evaluated using chicken meat samples, showing its potential applicability to rapidly detect BAs produced inside the packaging. Moreover, after improving its stability, the developed sensor could be, in the near future, commercialized as a smart label to detect food spoilage inside packaging. Therefore, ongoing efforts are focused on improving the sensor’s stability, for example, by using edible polymers to integrate genipin [40]. In addition, other sensors responding to different BAs could be integrated to achieve multiplexed detection. We believe that the proposed sensor could represent a facile tool for the food packaging industry to design innovative smart packaging for the benefits of suppliers, retailers and consumers.

## Data Availability

The data presented in this study are available on request from the corresponding author.

## Supplementary Materials

The following supporting information can be downloaded at: https://www.mdpi.com/article/10.3390/bios13010126/s1, Figure S1: Sensor responsiveness at 18 h in liquid format; Figure S2: Picture and data elaboration of sensor responsiveness in a 96-microtiter plate at 3 h; Figure S3: Putrescine calibration curves obtained with genipin adsorbed and entrapped on paper.
