# Supplementary material for: Colorimetric Paper Sensor for Food Spoilage Based on Biogenic Amine Monitoring
Source: Biosensors (Basel). 2023 Jan 11;13(1):126. doi: 10.3390/bios13010126 (PMC9855854; doi:10.3390/bios13010126)
Supplement: Supplementary file 1 [file biosensors-13-00126-s001.zip › biosensors-2144884-supplementary.pdf]

Supplementary Materials

Colorimetric paper sensor for food spoilage based on biogenic amines monitoring

Maria Maddalena Calabretta [1,2], Denise Gregucci[1,2], Riccardo Desiderio[1] and Elisa Michelini[1,2,3*]

[1]Department of Chemistry "Giacomo Ciamician", Alma Mater Studiorum - University of Bologna, Via Selmi 2, 40126, Bologna, Italy

[2]Center for Applied Biomedical Research (CRBA), Azienda Ospedaliero-Universitaria Policlinico S. Orsola-Malpighi, Bologna, Italy

[3]Health Sciences and Technologies Interdepartmental Center for Industrial Research (HSTICIR), University of Bologna, 40126 Bologna, Italy

*Corresponding author:

Prof. Dr. Elisa Michelini

Departement of Chemistry "Giacomo Ciamician"

University of Bologna

Via Selmi 2, 40126 Bologna, Italy

Tel.: +39 051 20 9 9533

e-mail: elisa.michelini8@unibo.it

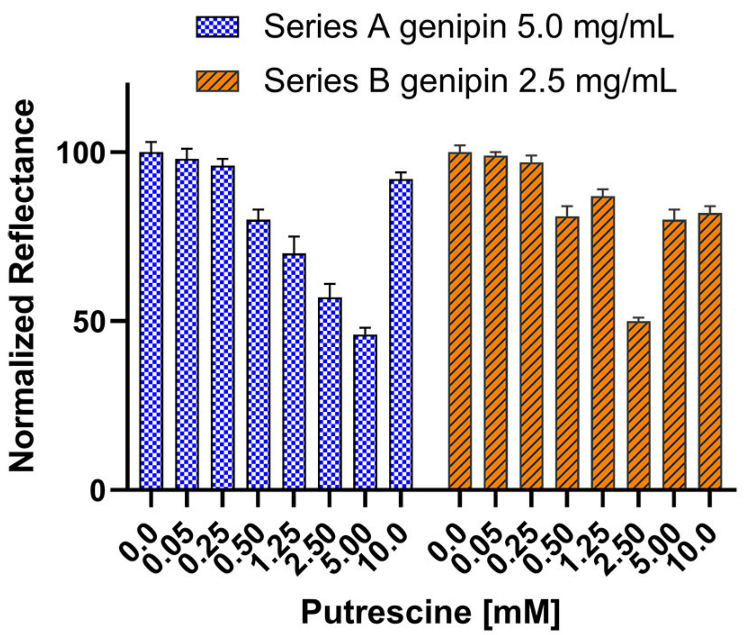

Figure S1: Sensor responsiveness in liquid format obtained in clear microtiter 96-well plate adding 40 μL of 5.0 mg/mL genipin (series A) or 2.5 mg/mL (series B) solution and a 40 μL volume of putrescine (concentration range from 0 to 5 mM) and incubated at 25°C for 18 h.

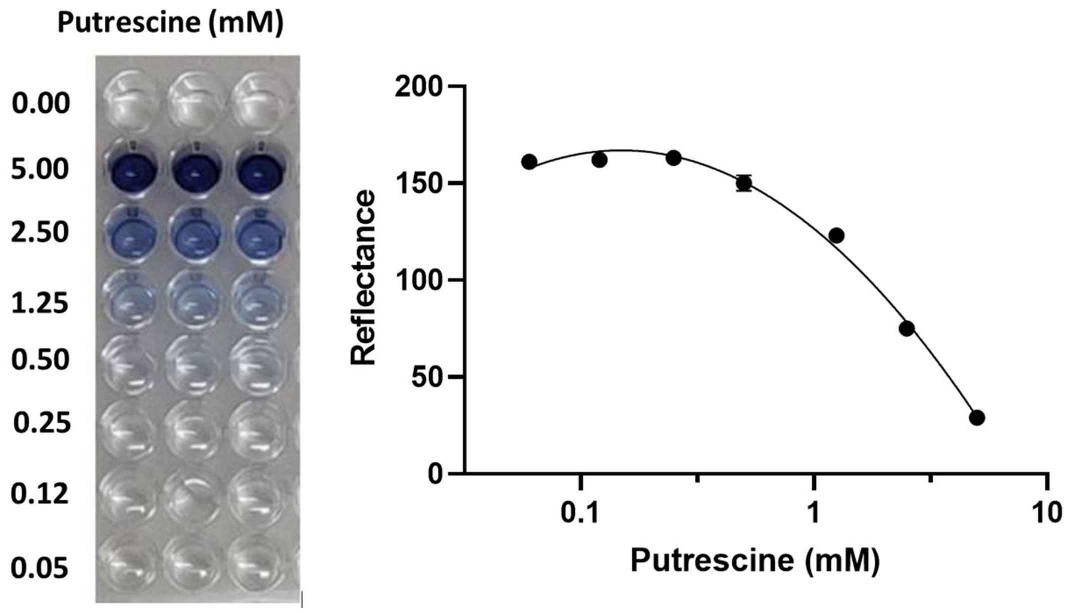

Figure S2: Picture and data elaboration of sensor responsiveness in liquid format in 96-microtiter plate obtained adding 40 μL of 5.0 mg/mL genipin and a 40 μL-volume of putrescine (concentration range from 0 to 5 mM) and incubated at 25°C for 3 h.

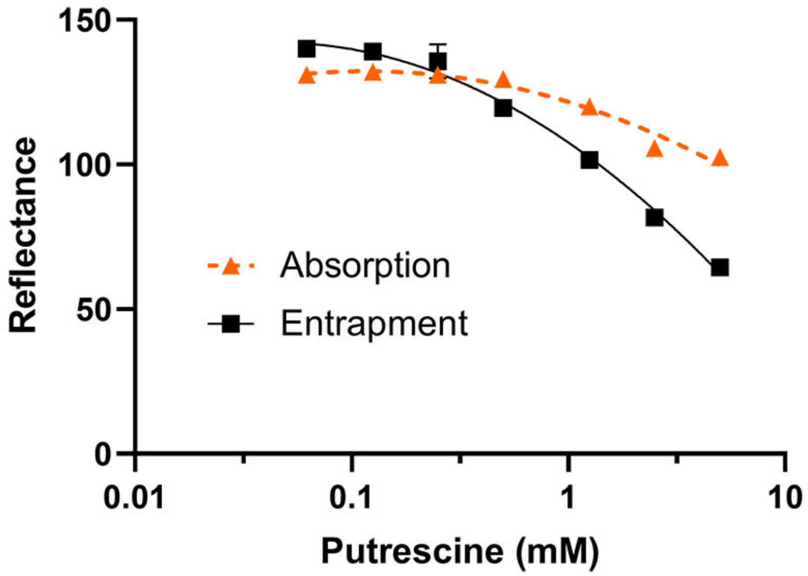

Figure S3: Putrescine calibration curves obtained with genipin (5.0 mg/mL) adsorbed and entrapped on paper, after 3 h of incubation at +25°C.
